# Chemical data on ashy soils as an information basis for dating archaeological sites

**DOI:** 10.1016/j.dib.2020.106691

**Published:** 2020-12-24

**Authors:** Fedor N. Lisetskii, Arseniy O. Poletaev, Vladimir F. Stolba

**Affiliations:** a Federal and Regional Centre for Aerospace and Surface Monitoring of the Objects and Natural Resources, Belgorod National Research University, Belgorod 308015, Russian Federation; b Institute of Earth Sciences, Belgorod National Research University, Belgorod 308015, Russian Federation; c Berlin-Brandenburgische Akademie der Wissenschaften, Jägerstrasse 22/23, D-10117 Berlin, Germany

**Keywords:** Archaeological soils, Ash, Ash Deposits, Fuel, Pedoarchaeology, Geoarchaeology, Geochemistry

## Abstract

As a special type of parent rock associated with human activities both in antiquity and nowadays, ash widely occurs in the settlements’ functional zones and their cultural layers. Soils developed on ash deposits of various genesis can be presented as soil chronosequences, which forms an information basis for determining the time at which settlements and their economic zones went out of use (“Archaeological ash deposits and soils formed on ash in the south of the East European Plain. Quaternary International” [1]). Studies of ash deposits and soils formed on ash were conducted in three regions of the East European Plain which differ in extent of forest cover. Geochemical associations of accumulated and dispersed elements in the upper horizon of soils of different age in relation to the original ash were determined. This makes it possible to calculate the time of biogeochemical transformation of ash in the course of pedogenesis, thus offering a new dating technique for archaeologists.

## Graphical abstract

**Figure:**
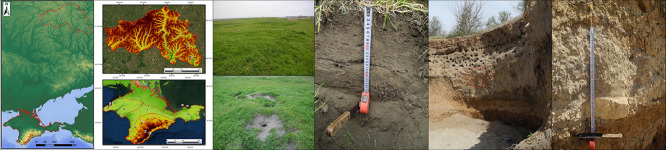

**Specifications Table**

**Table:** 

Subject	Soil Science
Specific subject area	Pedoarchaeology, Geochemistry
Type of data	Table Image Figure
How data were acquired	XRF spectrometer (Spectroscan Max-GV, ‘SPECTRON’, Ltd). Emission spectrometer ICPE–9000 with induction-bound plasma (Zn, Cu, Mn and Co), atomic absorption spectrometer Quant–2AT (Pb and C), spectrometer Quant–Z (Hg), photocolorimeter KFK–3–01 (As). Method of cluster analysis (hierarchic classification, unification by Ward's method). STATISTICA Advanced + QC for Windows v.10 Ru.
Data format	Raw
Parameters for data collection	The data derives from our study of 14 archaeological sites in the south of the East European Plain. The chemical composition of sampled ash deposits and soils formed on ash, as well as of plant samples (fossil and modern oak and grass plants), was analysed by 22 chemical elements, including 10 macroelements and 12 trace elements.
Description of data collection	The dataset includes the information on specific features of archaeological ash in relation to three other types of principal parent materials, on chemical properties of soils formed on ash, biogeochemical properties of soil formed on ash in the region with significant proportion of oak forests both in antiquity and today, and on the values of informative geochemical indicators for ash groups of different genesis for the regional reconstruction of palaeogeographic conditions.
Data source location	Belgorod region, Russian Federation: Borisovka, mid-5th c. BC (50°37′15.50"N; 36°0′13.40"E). Crimean Peninsula, Russian Federation: Kalos Limen, 4th c. BC – 2nd c. AD (45°31′2.90"N; 32°42′56.90"E); former village of Saya, before 1944 (45°31′7.95"N; 32°49′14.48"E); Chernomorskoe/Settlement S11-022, Bronze Age and Early Iron Age (45°29′21.10"N; 32°43′17.49"E); ancient Greek farmhouse of Kunan, 2nd c. BC (45°25′49.62"N; 32°41′35.52"E); former village of Ojrat, before 1944 (45°19′36.18"N; 32°40′14.52"E); Vitino, 4th – 3rd c. BC (45°14′10.07"N; 33°8′13.20"E); Airchi, 2nd c. BC – 1st c. AD (45°12′6.28"N; 33° 9′8.45"E). Ak-Kaya, 225–250 AD (45°7′20.04"N; 34°36′11.58"E); Borut-Khane, 1st c. BC – 1st c. AD (45°6′7.56"N; 34°18′32.76"E); Kermen-Kyr, 2nd – 3rd c. AD (44°58′39.48"N; 34°3′29.34"E); Zayachye, 2nd – 3rd c. AD (44°51′30.90"N; 33°48′50.28"E); settlement of Mysovka (Mysovoe II), 4th c. BC – 3rd c. AD (45°26′56.39"N; 35°49′16.69"E); Kazantip Vostochny 1, 3rd c. BC – 3rd c. AD (45°26′57.69"N; 35°50′29.94"E).
Data accessibility Related research articles	Data provided within this article F.N. Lisetskii, V.F. Stolba. Archaeological ash deposits and soils formed on ash in the south of the East European Plain. Quaternary International (2021). 10.1016/j.quaint.2020.11.030

**Value of the Data**

• Geochemical differences in soils formed on ash, which is reflected in an increased concentrations of Cu, As, SiO_2_, Al_2_O_3_ and Pb and decreased concentrations of Sr, Ca, Co, Mg and Na_2_O, form the information basis for dating the termination of activities at archaeological sites and their economic zones.
• In regions with different sources of available fuel (wood in the forest-steppe and dung in the steppe zone), fossil ash can aid the reconstructions of paleoclimate and living conditions, using the ensemble of chemical elements (P, K, Mn, Zn, Co, Pb, Sr and Zr) as an indicator.
• Comparison of the chemical composition of experimentally ashed fossil oak wood and forb-cereal hay made it possible to determine the elements indicative of wood ash and of ash resulting from the combustion of other fuels available in the steppe conditions, which makes it possible to ascertain the genesis of archaeological ash deposits.

## 1 Data Description

Geochemical differences between the main parent rocks and ash and soils formed on ash (by 6 macroelements and 6 trace elements, as well as by the SiO_2_/(RO+R_2_O) ratio) reflect the specificity of ash as a parent material [1]. The hierarchical classification of ash and soils formed on ash by 12 most informative chemical elements (Sr, Ca, Co, Mg, Na, Cu, As, Si, Al, Pb, K, P) has made it possible to observe the geochemical transformation of ash in ashy deposits in the course of pedogenesis. Geochemical features of ash deposits and soils formed on ash for three regions that differ in shares of forest cover are presented in the form of input data and calculated coefficients. Oak wood was obtained from the excavations of burial mound Ak-Kaya 9, where 2325-2350 years ago, at a depth of 9.5 m, it has been used in the floor construction. As various plants and other fuels differ in their elemental composition, chemical analysis of ash is an important tool for identifying different ancient fuels [2]. In addition, experimental ashing of fossil wood (*Quercus pubescens*) and hay from steppe grasses with subsequent chemical analysis has been carried out to identify archaeological ashes obtained from various types of fuel.

**Fig. 1:**
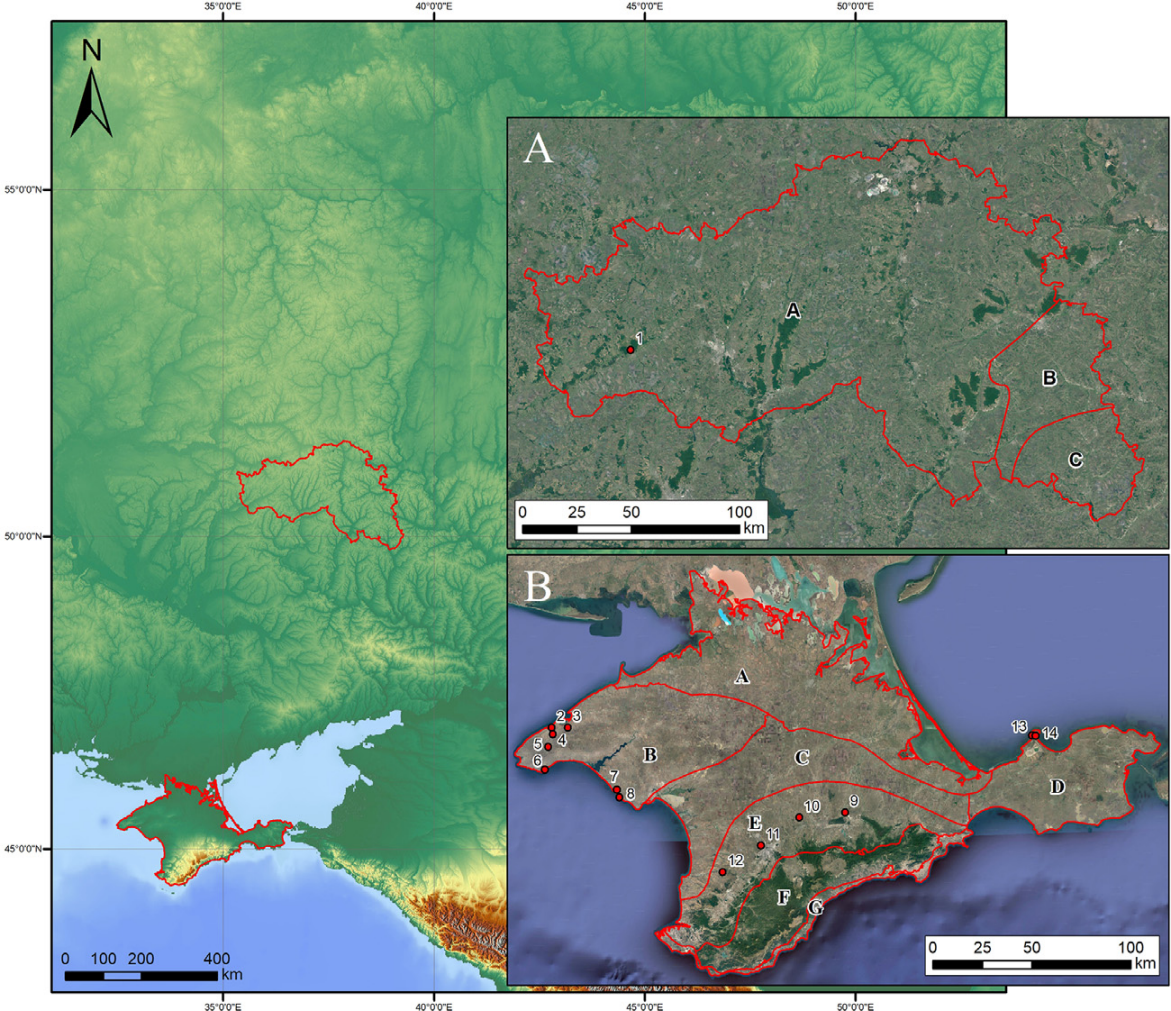
Location of research objects (ashy soil and ash deposits) within the continental forest-steppe, Belgorod Oblast (A) and within the Plain (steppe) and Piedmont (forest-steppe) Crimea (B). Archaeological sites: Borisovka, mid-5th c. BC (1); Kalos Limen, 4th c. BC – 2nd c. AD (2); former village of Saya, before 1944 (3); Chernomorskoe/Settlement S11-022, Bronze Age and Early Iron Age (4); Kunan, 2nd c. BC (5); Former village of Oirat, before 1944 (6); Vitino 4th – 3rd c. BC (7); Airchi, 2nd c. BC – 1st c. AD (8); Ak-Kaya, c. 225–250 AD (9); Borut-Khane, 1st c. BC – 1st c. AD (10); Kermen-Kyr, 2nd – 3rd c. AD (11); Zayachye, 2nd – 3rd c. AD (12); Settlement of Mysovka (Mysovoe II), 4th-2nd c. BC – 3rd c. AD (13); Kazantip Vostochny 1, 3rd – 2nd c. BC – 3rd c. AD (14). Natural areas (A): A – Typical forest-steppe; B – Southern forest-steppe; C – Steppe. Natural areas (B): A – North Crimean Lowland steppe; B – Tarkhankut elevated plain; C – Central Crimean Plain steppe; D – Kerch hilly-ridged steppe; E – Foothill forest-steppe; F – Main ridge, mountainous meadows and forests; G – Southern Coast. A screenshot of the Relief map was downloaded from the site: https://maps-for-free.com/

**Fig. 2:**
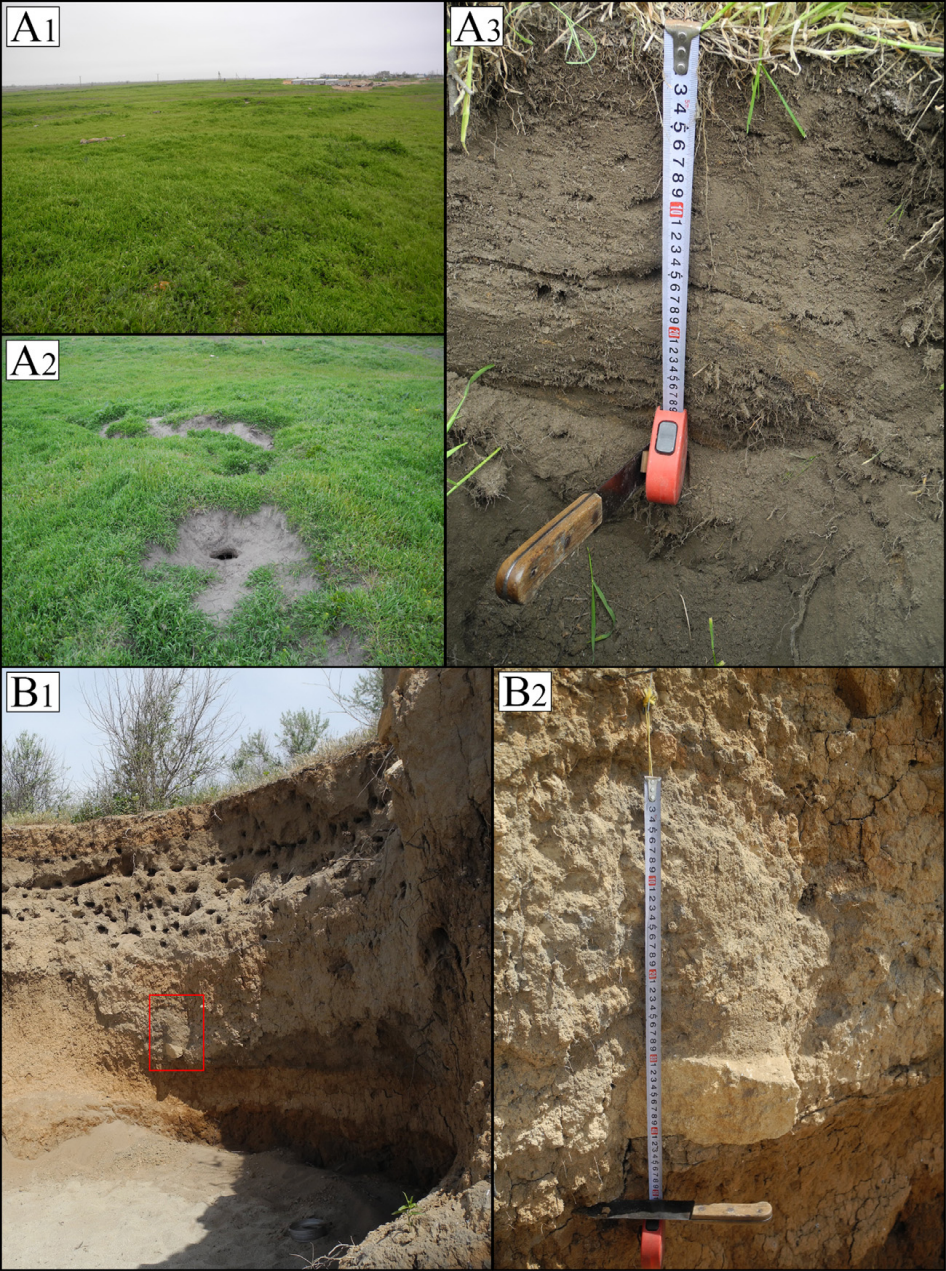
Location examples s of ash deposits on positive and negative relief elements in archaeological landscapes: Ash hill near the Tatar settlement of Oirat (A) 1784-1944 AD (A1 – panorama (center), A2 – hilltop, A3 – hilltop soil profile); Ash horizon at a depth of 180-213 cm in the fill of a 1st c. BC defensive ditch at the Airchi (B) settlement (B1 – ditch, B2 – ash horizon).

**Fig. 3:**
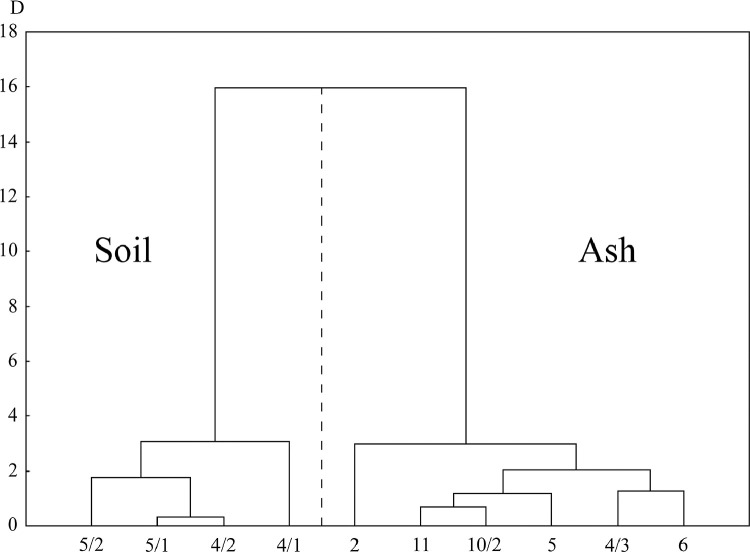
Dendrogram of the ash (right) and soil-on-ash (left) sample distribution. D - Threshold distance. *Archaeological sites: 5, 5/1, 5/2 – Kunan, ancient Greek farmhouse, 2nd c. BC; 4/1, 4/2, 4/3 – Chernomorskoe/Settlement S11-022 (Bronze Age and Early Iron Age); 2 – Kalos Limen (4th c. BC – 2nd c. AD), ash deposit; 11 – Kermen-Kyr, 2nd – 3rd c. AD; 10/2 – Borut-Khane (1st c. BC – 1st c. AD), ash deposit; 6 – ash deposit near the village of Oirat (before 1944).

**Fig. 4:**
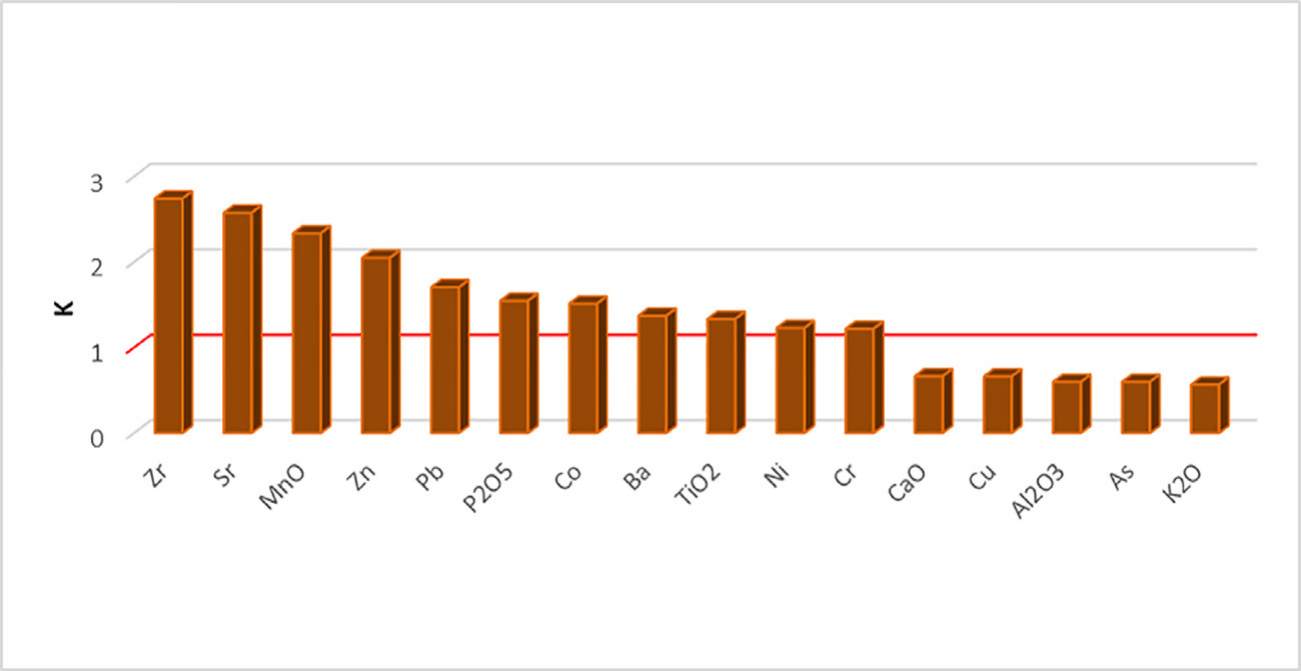
Correlation of the content of chemical elements in ash from archaeological sites of the forest area (No. 1) and sparse foothill zone (No. 10). Figure reflects К=C_i(1)_ / C_i(10)_ with differences greater than or less than 20%.

**Fig. 5:**
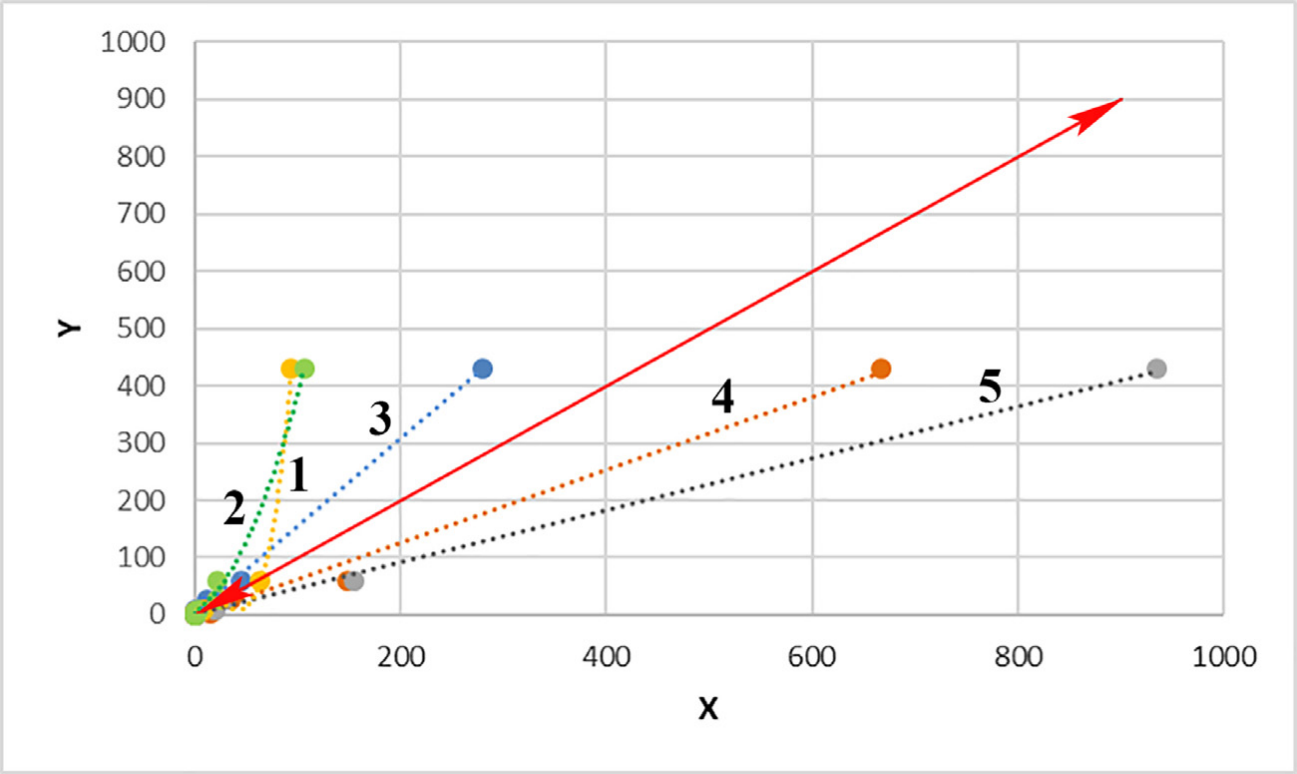
Regression dependences of the contents of Zn, Cu, Mn, Cd, Pb, As, Hg, Mo, and Co in the ash from the soil section at the settlement of Borut-Khane, 1st c. BC – 1st c. AD (Y) and in ash from samples of organic matter: modern oak (*Quercus pubescens*) (1); fossil oak (2); feather grass (*Stipa capillata*) (3); cow dung (4); horse manure (5).

Fig. 1 shows location of research objects (ashy soil and ash deposits) in the continental forest-steppe, Belgorod Oblast, and the Plain (steppe) and Piedmont (forest-steppe) Crimea. Table 1 lists objects of study at archaeological sites within three research regions (soils formed on ash and ash from cultural strata). Fig. 2 provides examples of the location of ash deposits on positive and negative relief elements in archaeological landscapes. Table 2 shows geochemical features of the main parent rocks, ash and soils formed on ash. Table 3 displays chemical properties of soils of archaeological sites in the south of the Kazantip Peninsula. Table 4 shows geochemical transformation of soil formed on ash (Borisovka settlement) over 24 centuries. Table 5 comprises the chemical composition of soils and ash of different ages at archaeological sites of the North-West Crimea. Table 6 contains the chemical composition of soils and ash at archaeological sites of the Piedmont Crimea. Fig. 3 features a dendrogram for the classification of ash and soils formed on ash deposits. Table 7 shows colouring and chemical composition of ash in groups classified by cluster analysis. Table 8 reports the main geochemical indicators of group objects (soils and ash), resulted from the cluster analysis. Table 9 compares the chemical composition of oak wood ash (OWA) and ash from hay of mixed grasses (GMA), using two ranked lists. Fig. 4 shows correlation of the content of chemical elements in archaeological ash from the forest area and poorly forested foothill zone. Fig. 5 shows regression dependences in the contents of Zn, Cu, Mn, Cd, Pb, As, Hg, Mo, and Co in the ash datable to the 1st c. AD and in experimentally ashed organic samples (modern oak; fossil oak; feather grass; cow dung and horse dung).

**Table 1:** Objects of study at archaeological sites from three research regions (soils formed on ash and ash from cultural strata).

No.	Soil/Ash	Depth (cm)	Soil colour (Munsell)		R_CIE_
			moist	dry	(dry)
1	Ash	100	10YR 5/2.5	10YR 6/2	0.442
2	Hor. А	0-22	10YR 5/2.5	10YR 6/2	0.442
2	cultural layer	>20	10YR 5/3	10YR 7/2	0.150
3	Hor. А	20	10YR 3/3	10YR 5/2.5	1.956
	cultural layer	50	10YR 4/3	10YR 5/3	2.393
4	Hor. А	0-18	10YR 3/3	10YR 5/2	1.519
	Hor. АВ	18-50	10YR 3/2	10YR 6/2	0.442
	Ash	>50	10YR 5/4	10YR 7/2	0.150
4	Hor. А	5-26	10YR 3/3	10YR 5/3	2.393
	Hor. АВ	26-55.5	10YR 3/3	10YR 5/2	1.519
	Hor. В1	47-55.5	10YR 3/3	10YR 5/2.5	1.956
	Ash	55.5-60.5	10YR 4/3	10YR 6/2.5	0.572
4	Hor. C	60–70	10YR 3/3	10YR 6/2	0.442
	Hor. С	80–90	10YR 4/3	10YR 6/2	0.442
5	39–41	39–41	10YR 3/2	10YR 5/1.5	1.139
5	Ash	>41	10YR 4/3	10YR 5/3	2.393
6	Hor. А	0–16	10YR 3/2	10YR 6/2	0.442
	Hor. АВ	16–28	10YR 4/3	10YR 6/2	0.442
7	Ash	> 30	10YR 4/2	10YR 6/2	0.442
8	235	235	10YR 5/2	10YR 7/1	0.075
8	cultural layer	200	10YR 5/3	10YR 6/2	0.442
8	ash on the hearth stone	152	10YR 4/4	10YR 6/4	0.924
9	Hor. В1	> 33.5	10YR 4/2	10YR 6/2	0.442
10	Hor. В1	> 37	10YR 5/3	10YR 6/2	0.442
10	Ash	63-73	10YR 5/3	10YR 7/1	0.075
11	Hor. В1	30-35	10YR 4/3	10YR 6/1	0.240
12	Hor. В1	> 32	10YR 5/3	10YR 6/1	0.240

**Table 2:** Initial geochemical data, their mean values and errors (Sx) for main parent rocks, ash and soils formed on it.

Point no.	Macro-elements (%)						Trace elements (ppm)						SiO_2_ / R_2_O_3_*
	CaO	P_2_O_5_	SiO_2_	Al_2_O_3_	Fe	MgO	Co	Cu	Pb	Sr	Zn	Ni	
Carbonate light brown loams (North-Western Crimea)													
1	27.9	0.2	24.6	7.0	1.9	2.8	4.8	11.5	9.1	243.5	56.6	28.8	2.7
2	22.5	0.2	20.4	7.1	1.8	2.3	7.1	19.1	10.9	157.7	61.4	29.2	2.3
3	23.9	0.2	18.1	6.8	1.8	2.5	6.3	21.2	8.7	160.9	57.3	30.2	2.1
4	23.1	0.2	40.0	9.2	2.4	2.7	8.1	26.9	13.5	182.9	74.9	39.9	3.4
5	26.0	0.3	26.9	9.3	2.5	2.6	5.5	28.0	16.5	109.2	67.5	35.4	2.3
6	24.4	0.3	21.2	7.5	2.5	2.6	5.7	17.4	17.3	135.1	70.2	32.6	2.1
7	26.9	0.2	26.5	9.4	2.3	2.9	7.8	23.2	10.7	242.1	73.8	37.2	2.3
8	28.0	0.1	31.0	8.7	2.0	3.0	6.2	15.0	8.8	212.4	65.3	33.3	2.9
9	22.2	0.1	33.3	8.3	2.2	2.7	8.6	18.0	12.1	251.5	65.1	33.9	3.2
10	22.7	0.1	35.9	8.6	2.1	2.4	8.0	25.0	6.9	221.8	65.2	37.0	3.4
11	21.4	0.2	31.4	7.9	2.2	2.2	11.4	22.6	11.7	227.7	61.4	36.0	3.1
Mean	24.5	0.2	28.1	8.2	2.2	2.6	7.2	20.7	11.5	195.0	65.3	34.0	2.7
S_x_	0.72	0.02	2.07	0.29	0.07	0.08	0.55	1.53	0.99	14.61	1.82	1.08	0.15
Carbonate brown and grayish-brown loams, underlain by limestone (North-Western Crimea)													
1	10.6	0.1	44.4	10.2	2.9	1.9	16.4	44.3	17.5	234.9	76.1	51.2	3.4
2	11.7	0.2	46.3	10.9	3.0	2.1	12.9	42.3	19.2	318.9	74.3	48.8	3.3
3	13.8	0.5	36.9	10.4	2.8	2.1	11.3	37.3	19.9	202.0	81.6	43.5	2.8
4	13.8	0.6	36.9	10.4	2.8	2.0	12.4	31.8	16.6	202.4	91.3	43.1	2.8
5	8.4	0.1	40.3	9.3	3.0	1.2	16.4	51.3	20.4	127.9	72.4	51.8	3.2
6	14.1	0.2	40.7	8.8	2.6	2.0	14.2	33.5	16.8	232.6	65.0	42.2	3.5
7	9.9	0.1	46.6	9.3	2.8	1.4	13.5	37.1	19.0	181.9	64.0	43.2	3.8
8	11.5	0.4	35.7	9.1	3.1	1.8	8.7	36.4	17.0	121.3	72.9	39.7	2.9
9	11.2	0.3	48.1	9.3	2.6	1.7	10.9	33.9	17.3	207.8	74.4	40.5	4.0
Mean	11.7	0.3	41.8	9.7	2.8	1.8	13.0	38.7	18.2	203.3	74.7	44.9	3.3
S_x_	0.65	0.06	1.58	0.25	0.06	0.11	0.84	2.08	0.48	19.75	2.75	1.51	0.15
Ash from the cultural layer of the archaeological sites of Crimea													
1	15.6	1.2	28.0	5.7	2.0	2.3	19.1	18.2	13.1	349.6	123.4	34.7	3.6
2	18.0	1.3	39.2	8.9	2.7	2.4	16.4	27.0	19.9	359.9	147.5	44.8	3.3
3	20.4	1.2	24.1	6.3	2.3	2.6	15.3	18.1	28.2	367.4	119.4	33.6	2.8
4	16.5	1.1	46.6	7.2	2.3	2.6	17.7	20.7	18.6	338.9	124.8	45.4	4.8
5	17.7	1.4	35.9	7.6	2.2	2.3	10.4	19.2	16.1	304.4	133.2	36.7	3.6
6	19.0	1.2	36.2	7.4	2.3	2.2	17.5	19.0	19.8	376.3	145.6	36.8	3.7
7	16.4	0.7	40.2	6.9	2.3	1.7	15.7	17.2	16.6	275.5	106.4	37.9	4.3
8	15.3	0.4	45.0	9.3	2.3	2.1	20.0	48.2	14.0	683.8	52.2	48.6	3.8
9	13.1	0.4	51.8	10.2	2.4	2.0	16.5	42.6	13.7	563.7	71.0	48.8	4.1
10	25.1	1.2	38.7	7.3	1.5	2.6	7.1	46.4	20.1	289.4	60.4	41.4	4.4
11	10.2	0.6	54.4	9.0	2.4	1.7	15.7	35.0	12.5	149.9	105.7	44.7	4.7
12	13.6	0.7	47.0	8.6	2.2	1.6	15.3	46.8	11.5	175.3	82.8	47.6	4.3
Mean	16.7	0.96	40.6	7.9	2.2	2.2	15.6	29.9	17.0	352.8	106.0	41.7	4.0
S_x_	1.10	0.10	2.60	0.39	0.08	0.10	1.03	3.74	1.35	42.65	9.38	1.62	0.17
Soils formed on ash													
1	9.1	1.0	52.9	9.0	2.7	1.4	15.1	49.4	20.8	176.4	174.6	57.6	4.5
2	11.9	1.1	51.9	8.8	2.7	1.7	16.4	34.8	18.9	207.4	166.4	48.5	4.5
3	6.0	0.6	53.5	8.9	2.8	1.2	16.5	40.0	24.5	162.2	116.4	46.2	4.5
4	8.9	0.6	55.0	9.1	2.8	1.5	16.5	34.7	21.6	176.9	167.1	44.8	4.6
5	13.8	1.1	30.6	6.6	2.2	1.9	13.4	29.8	17.2	234.7	126.6	37.1	3.5
6	17.9	1.2	36.8	5.9	2.1	3.3	18.0	11.7	33.4	531.3	113.6	39.5	4.5
7	16.9	1.3	45.8	7.6	2.2	2.5	8.6	20.8	38.1	402.2	143.8	36.3	4.7
8	7.0	0.5	55.2	10.3	2.6	1.6	20.5	49.6	13.8	132.2	80.3	52.3	4.3
9	8.3	0.6	42.0	7.6	2.9	1.3	14.2	45.0	17.8	202.6	106.1	48.9	3.9
Mean	11.1	0.89	47.1	8.2	2.5	1.8	15.5	35.1	22.9	247.3	132.8	45.7	4.3
S_x_	1.44	0.11	2.96	0.46	0.11	0.23	1.11	4.28	2.65	43.92	10.75	2.37	0.13

⁎ SiO_2_ / R_2_O_3_ = SiO_2_ / ∑(Al_2_O_3+_ Fe_2_O_3_+ MnO).

**Table 3:** Chemical properties of soils of archaeological sites in the south of the Kazantip Peninsula.

Object of study	Data	Horizon, depth (cm)	Chemical indicators			
			CaCO_3_*, %	Corg, %	Total N, %	С:N*
Settlement of Kazantip Vostochny 1	3rd-2nd c. BC – 3rd c. AD	А, 3-21	14.89	4.17	0.487	8.6
		АВ, 21-40	17.59	2.11	0.330	6.4
		С, >40	18.61	1.88	0.182	10.3
Ash deposit 150 m west of the settlement of Kazantip Vostochny 1	3rd-2nd c. BC – 3rd c. AD	А, 6-17	11.50	3.91	0.448	8.7
		С, >45	18.61	2.14	0.226	9.5
Settlement of Mysovka (Mysovoe II)	4th-2nd c. BC – 3rd c. AD	А, 0-22	13.68	3.52	0.400	8.8
		С, 28-32	20.22	1.67	0.204	8.2

⁎ The scale [8] suggests that degree of humus enrichment with nitrogen (C:N) is high (C:N is 5–8), and medium (C:N is 8–11).

**Table 4:** 24-century long geochemical transformation of soil formed on ash (Borisovka settlement).

	Newly formed soil			Ash
Layer, cm	A, 0-23	AB, 23-41	B, 41-57	C, 62-72
Soil color (dry)	10YR 5/2.5	10YR 5/2	10YR 6/1.5	10YR 6/2
Macroelements, %				
SiO_2_	59.57	61.24	55.48	32.05
Al_2_O_3_	7.09	7.85	7.53	4.51
CaO	2.69	7.01	14.85	11.74
Fe	2.05	1.97	1.82	2.51
TiO_2_	0.89	0.84	0.65	0.60
P_2_O_5_	0.80	1.90	3.26	2.18
K_2_O	1.68	1.70	1.51	1.10
MnO	0.19	0.22	0.17	0.21
MgO	0.90	1.36	2.15	2.28
Na_2_O	1.14	1.31	1.96	2.81
Trace elements, mg kg^−1^				
As	6.12	3.49	4.08	2.78
Ba	631.85	640.68	652.06	692.97
Co	11.91	11.78	19.33	15.76
Cu	32.26	18.48	2.32	12.70
Cr	95.61	72.45	69.92	88.94
Ni	35.39	27.34	28.38	44.98
V	69.49	58.54	53.94	48.52
Pb	20.19	19.83	12.32	27.37
Rb	78.47	66.51	58.43	68.32
Sr	236.22	326.22	593.16	783.79
Zn	143.84	175.44	190.18	272.84
Zr	505.59	409.81	353.61	444.15

**Table 5:** The geochemical composition of soils and ash of different age at archaeological sites of North-West Crimea.

Objects*	Depth, cm	Sr	CaO	Co	MgO	Na_2_O	Cu	As	SiO_2_	Al_2_O_3_	Pb	K_2_O	P_2_O_5_	SiO_2_/(RO+R_2_O)⁎⁎
		ppm	%	ppm	%	%	ppm	ppm	%	%	ppm	%	%	dimensionless
Soils of different age on ash substrate														
4/2	5-26	132.16	6.97	20.52	1.58	1.17	49.65	7.57	55.20	10.25	13.81	1.90	0.52	4.75
	26-55.5	202.63	8.32	14.16	1.27	1.07	44.96	4.39	42.00	7.63	17.82	1.69	0.55	3.40
	47-55.5	174.61	10.78	13.52	1.55	1.32	32.06	6.59	48.93	8.77	15.91	1.82	0.64	3.16
4/1	0-18	162.22	5.99	16.52	1.18	0.83	40.02	4.70	53.54	8.90	24.54	1.84	0.56	5.44
	18-50	176.88	8.85	16.50	1.45	1.12	34.69	4.64	55.02	9.08	21.60	1.86	0.61	4.14
5/1	0-19	176.43	9.05	15.10	1.38	1.12	49.38	5.13	52.90	8.99	20.76	1.94	1.04	3.92
	19-38	207.42	11.86	16.41	1.65	1.42	34.84	5.25	51.85	8.80	18.91	1.85	1.11	3.09
2/1	0-22	531.26	17.91	17.99	3.28	2.27	11.72	10.17	36.81	5.86	33.39	2.32	1.15	1.43
6	0-28	402.24	16.94	8.56	2.50	1.32	20.82	7.74	45.80	7.57	38.05	3.00	1.33	1.93
3	20	162.07	16.48	8.10	1.92	1.19	42.10	7.43	34.01	8.14	< LOQ	1.83	0.49	1.59
	50	178.75	14.35	10.32	1.55	1.36	33.83	6.17	36.39	7.73	14.99	1.77	0.53	1.91
Ash from cultural strata of archaeological sites														
5/1	39-40	338.90	16.49	17.65	2.56	2.19	20.65	5.07	46.63	7.19	18.55	2.02	1.10	2.00
4/1	>50	275.45	16.38	15.67	1.71	1.47	17.15	5.63	40.23	6.87	16.56	1.67	0.69	1.89
2/2	>20	289.39	25.08	7.13	2.57	2.19	46.41	4.77	38.68	7.28	20.15	1.56	1.20	1.23
4/2	55.5-60.5	231.66	14.06	13.49	1.90	1.51	22.21	6.79	47.26	8.03	6.45	1.78	0.73	2.45
4/3	70-80	149.86	10.25	15.69	1.67	1.39	35.02	5.52	54.42	9.01	12.45	1.99	0.65	3.56
4/3	90-100	175.32	13.64	15.34	1.59	1.47	46.81	5.81	46.96	8.64	11.46	1.83	0.70	2.53
8/1	235	683.84	15.33	19.99	2.15	1.64	48.18	4.25	44.95	9.34	13.95	1.67	0.44	2.16
8/2	200	563.74	13.13	16.53	2.03	1.64	42.62	4.95	51.81	10.21	13.66	1.90	0.43	2.77
8/2	152	916.93	24.43	8.40	2.80	2.06	15.42	5.19	37.22	8.51	13.54	1.53	0.54	1.21
7	0-22	465.65	15.54	16.20	3.05	2.39	26.15	30.74	44.55	7.85	68.57	3.16	1.18	1.85
Soil average (S)														
Average		227.88	11.59	14.34	1.76	1.29	35.82	6.34	46.59	8.34	21.98	1.98	0.78	3.16
Ash average (A)														
Average⁎⁎		409.07	16.43	14.61	2.20	1.80	32.06	7.87	45.27	8.29	19.53	1.91	0.77	2.17
Percentage of soil differences in relation to ash (100•[(S/A)-1])														
%		-44.29	-29.47	-1.87	-20.32	-28.13	11.74	-19.42	2.91	0.54	12.51	3.80	1.23	45.96

⁎ Archaeological sites: 4/1, 4/2, 4/3 – Chernomorskoe/S11-022 (Bronze Age); 5/1 - Kunan, ancient Greek farmhouse; 2/1 - Kalos Limen, ash deposit; 6 - ash deposit near the village of Oirat; 3 - ash deposit near the village of Saya; 2/2 - Kalos Limen, the citadel; 8/1 - Archi, defensive ditch; 8/2 - Archi, pit at the settlement; 8/2 - Archi, late Scythian furnace.

⁎⁎ RO = CaO+MgO; R_2_O = K_2_O + Na_2_O. ** Average calculated without the Airchi furnace.

**Table 6:** Geochemical composition of soils and ash at archaeological sites of the Piedmont Crimea.

Depth, cm	Sr	CaO	Co	MgO	Na_2_O	Cu	As	SiO_2_	Al_2_O_3_	Pb	K_2_O	P_2_O_5_	SiO_2_/(RO+R_2_O)*
	ppm	%	ppm	%	%	ppm	ppm	%	%	ppm	%	%	dimensionless
Borut-Khane, ancient settlement (1st c. BC - 1st c. AD)													
0-19	234.71	13.84	13.44	1.88	1.89	29.80	5.21	30.63	6.59	17.21	1.56	1.12	1.60
19-37	310.30	17.49	14.59	2.26	2.20	20.86	5.27	27.43	5.68	16.84	1.55	1.20	1.17
>37	349.58	15.57	19.07	2.28	2.04	18.23	6.10	28.02	5.72	13.07	1.81	1.22	1.29
Borut-Khane, ash deposit (1st c. BC - 1st c. AD)													
63-73	304.39	17.71	10.42	2.27	2.03	19.22	4.66	35.87	7.56	16.07	1.95	1.41	1.50
Ak-Kaya (Vishennoe), ancient settlement (3rd century AD)													
0-22.5	253.45	11.65	15.51	1.97	1.76	38.97	4.94	39.58	8.80	21.13	1.89	1.19	2.29
22.5-33.5	338.66	15.56	16.55	2.28	2.42	28.90	4.92	37.60	8.68	18.59	1.75	1.26	1.71
>33.5	359.92	17.96	16.43	2.35	2.47	26.97	3.51	39.17	8.93	19.94	1.79	1.30	1.59
Kermen-Kyr, ancient settlement (3rd century AD)													
0-12.5	233.53	11.21	16.04	1.68	1.71	27.06	6.48	42.17	8.52	26.83	1.66	1.05	2.59
12.5-21	296.11	14.67	17.44	2.16	2.12	23.97	5.76	39.15	8.14	19.16	1.65	1.14	1.90
30-35	376.31	19.00	17.54	2.18	2.21	18.95	4.10	36.21	7.37	19.84	1.73	1.23	1.44
Zayachye, eastern settlement (3rd century AD)													
0-22	202.15	10.45	11.44	1.74	2.02	27.40	8.21	40.47	8.56	29.13	1.64	1.17	2.55
22-30	288.39	18.23	10.14	2.17	2.30	18.21	8.20	32.61	7.30	24.87	1.58	1.24	1.34
>32	367.43	20.44	15.27	2.57	2.46	18.09	7.50	24.12	6.27	28.15	1.55	1.21	0.89
Soil average (0-35(37) cm) (S)													
	269.66	14.14	14.39	2.02	2.05	26.90	6.12	36.20	7.79	21.72	1.66	1.17	1.82
Ash average (A)													
	351.53	18.14	15.75	2.33	2.24	20.29	5.17	32.68	7.17	19.41	1.76	1.27	1.34
The percentage of soil differences in relation to ash (100•[(S/A)-1])													
%	-23.3	-22.1	-8.6	-13.6	-8.4	32.6	18.3	10.8	8.6	11.9	-6.0	-7.8	35.8

⁎ For each archaeological site, the termination date is indicated according to [9], as well as the dating of ceramics found when was the pit laying.

**Table 7:** Colour and chemical composition of ash in the groups identified by the cluster analysis.

Objects				Group А						x±S_x_			Group В						x±S_x_		
		1	4/1	5/2	4/2	4/2	4/2	8/1	8/2				10/1	9	12	5/1	10/2	11			
depth (cm)		62–72	>50	>41	60–70	80–90	55.5-60.5	200	152				>37	>33.5	>32	39-40	63-73	30-35			
Soil color (dry)		10 YR 6/2	10 YR 7/2	10 YR 5/3	10 YR 6/2	10 YR 6/2	10 YR 6/2.5	10 YR 7/1	10 YR 6/2				10 YR 6/2	10 YR 6/2	10 YR 6/1	10 YR 5/1.5	10 YR 7/1	10 YR 6/1			
TiO_2_	%	0.60	0.59	0.77	0.65	0.55	0.57	0.59	0.62	0.62	±	0.02	0.41	0.50	0.42	0.51	0.45	0.45	0.46	±	0.02
CaO	%	11.74	13.69	8.55	10.25	13.64	14.06	15.33	13.13	12.55	±	0.79	15.57	17.96	20.44	16.49	17.71	19.00	17.86	±	0.71
Al_2_O_3_	%	4.51	6.15	11.91	9.01	8.64	8.03	9.34	10.21	8.48	±	0.81	5.72	8.93	6.27	7.19	7.56	7.37	7.17	±	0.45
MnO	%	0.21	0.13	0.11	0.11	0.09	0.10	0.11	0.10	0.12	±	0.01	0.09	0.08	0.08	0.11	0.09	0.09	0.09	±	0.00
Fe	%	2.51	3.02	2.89	2.44	2.16	2.40	2.25	2.41	2.51	±	0.11	2.00	2.69	2.25	2.33	2.18	2.30	2.29	±	0.09
SiO_2_	%	32.05	33.89	57.66	54.42	46.96	47.26	44.95	51.81	46.13	±	3.23	28.02	39.17	24.12	46.63	35.87	36.21	35.00	±	3.28
P_2_O_5_	%	2.18	0.65	0.58	0.65	0.70	0.73	0.44	0.43	0.80	±	0.20	1.22	1.30	1.21	1.10	1.41	1.23	1.25	±	0.04
K_2_O	%	1.10	1.50	2.06	1.99	1.83	1.78	1.67	1.90	1.73	±	0.11	1.81	1.79	1.55	2.02	1.95	1.73	1.81	±	0.07
MgO	%	2.28	2.50	2.18	1.67	1.59	1.90	2.15	2.03	2.04	±	0.11	2.28	2.35	2.57	2.56	2.27	2.18	2.37	±	0.07
Na_2_O	%	2.81	2.89	1.90	1.39	1.47	1.51	1.64	1.64	1.91	±	0.21	2.04	2.47	2.46	2.19	2.03	2.21	2.23	±	0.08
Co	ppm	15.76	34.15	20.02	15.69	15.34	13.49	19.99	16.53	18.87	±	2.33	19.07	16.43	15.27	17.65	10.42	17.54	16.06	±	1.24
Ni	ppm	44.98	53.92	48.63	44.66	47.58	39.12	48.64	48.84	47.05	±	1.52	34.67	44.81	33.62	45.39	36.65	36.82	38.66	±	2.10
Cu	ppm	12.70	23.87	35.13	35.02	46.81	22.21	48.18	42.62	33.32	±	4.50	18.23	26.97	18.09	20.65	19.22	18.95	20.35	±	1.38
Zn	ppm	272.84	157.73	120.83	105.68	82.84	106.08	52.18	70.98	121.15	±	24.48	123.39	147.45	119.44	124.75	133.22	145.58	132.31	±	4.86
Sr	ppm	783.79	431.63	170.78	149.86	175.32	231.66	683.84	563.74	398.83	±	89.60	349.58	359.92	367.43	338.90	304.39	376.31	349.42	±	10.48
Pb	ppm	27.37	35.05	22.09	12.45	11.46	6.45	13.95	13.66	17.81	±	3.37	13.07	19.94	28.15	18.55	16.07	19.84	19.27	±	2.07
As	ppm	2.78	5.27	7.12	5.52	5.81	6.79	4.25	4.95	5.31	±	0.49	6.10	3.51	7.50	5.07	4.66	4.10	5.16	±	0.59
V	ppm	48.52	86.65	70.86	62.16	67.32	68.30	50.89	67.57	65.28	±	4.23	59.09	81.89	60.72	71.46	63.80	60.03	66.17	±	3.64
Ba	ppm	692.97	611.73	496.74	491.84	507.08	501.92	375.46	518.86	524.58	±	32.97	421.37	435.94	368.54	571.39	505.56	430.23	455.51	±	29.26
Cr	ppm	88.94	108.26	86.70	81.25	78.43	77.76	79.09	79.96	85.05	±	3.61	77.29	89.20	114.24	77.88	73.04	76.83	84.75	±	6.30
Zr	ppm	444.15	281.69	298.44	274.54	251.89	258.93	167.88	185.04	270.32	±	29.70	159.62	145.01	142.35	254.11	161.99	152.43	169.25	±	17.26
Rb	ppm	68.32	70.98	72.88	63.71	61.11	68.25	40.77	46.28	61.54	±	4.18	58.13	60.89	49.13	62.57	62.21	53.19	57.69	±	2.22

**Table 8:** Main geochemical indicators of the groups of objects (soils and ash) identified by the cluster analysis.

Object	Depth (cm)	CIA	(Ca+Mg+10•P)/Al	Ke	(Ca+Mg)/Al	Na/Al	Ca+Mg+K	Km	(Fe+Al)/ (Ca+Na+Mg)	Σ HM	SQ
1	100	18.54	7.94	1.77	3.11	0.62	15.12	8.71	0.42	420.39	7.15
4/1	>50	21.42	3.69	1.64	2.63	0.47	17.69	4.86	0.48	364.33	7.82
5/2	>41	45.58	1.39	3.90	0.90	0.16	12.79	2.20	1.17	291.89	8.39
4/2	60–70	36.31	2.04	3.53	1.32	0.15	13.91	2.03	0.86	255.61	7.71
4/2	80–90	29.65	2.57	2.52	1.76	0.17	17.06	1.87	0.65	240.69	7.52
4/2	55.5-60.5	27.55	2.90	2.44	1.99	0.19	17.74	2.35	0.60	232.78	7.25
8/1	200	28.86	2.34	2.15	1.87	0.18	19.15	1.28	0.61	217.64	7.66
8/2	152	33.74	1.91	2.76	1.48	0.16	17.06	1.48	0.75	228.70	7.91
10/1	>37	19.31	5.25	1.29	3.12	0.36	19.66	4.62	0.39	257.15	6.55
9	>33.5	24.35	3.73	1.59	2.27	0.28	22.10	3.93	0.51	303.50	7.95
12	>32	16.79	5.60	0.89	3.67	0.39	24.56	5.22	0.33	302.69	6.62
5/1	39-40	22.09	4.18	2.00	2.65	0.30	21.07	2.82	0.45	264.55	7.82
10/2	63-73	22.01	4.51	1.49	2.64	0.27	21.93	3.89	0.44	256.63	7.23
11	30-35	20.39	4.54	1.44	2.87	0.30	22.91	4.19	0.41	282.84	7.25
Average		26.19	3.76	2.10	2.31	0.29	18.77	3.53	0.58	279.96	7.49
Dispersion		60.33	2.90	0.68	0.57	0.02	11.34	3.66	0.05	2873.91	0.25
V. %		30	45	39	33	47	18	54	38	19	7

**Table 9:** Comparison of the chemical composition of oak wood ash (OWA) and ash from hay of mixed grasses (GMA) using two ranked lists.

Elements	Units	OWA	GMA	OWA / GMA		Elements	Units	GMA	OWA	GMA / OWA
As	ppm	3.96	< LOD	–		SiO_2_	%	75.84	< LOD	–
CaO	%	37.07	4.74	7.8		MnO	%	1.41	0.27	5.3
MgO	%	7.40	1.86	4.0		Zn	ppm	354.17	99.68	3.6
Sr	ppm	1571.57	516.41	3.0		Co	ppm	52.63	16.69	3.2
Na	%	4.71	1.64	2.9		P_2_O_5_	%	2.25	1.27	1.8
Zr	ppm	141.76	51.62	2.7		Ni	ppm	37.34	26.41	1.4
Al_2_O_3_	%	2.39	0.92	2.6		Pb	ppm	21.96	16.02	1.4
Fe	%	1.10	0.56	1.9						
Rb	ppm	28.60	15.15	1.9						
V	ppm	34.97	19.40	1.8						
Ba	ppm	805.92	541.31	1.5						
K_2_O	%	7.25	4.71	1.5						

*LOD is limit of detection.

## 2 Experimental Design, Materials, and Methods

### 2.1 Research objects

Objects of study (ashy soil and ash deposits) include 14 archaeological sites located within three research regions in the south of the East European Plain (Fig. 1, Table 1). Ash deposits can be found both on positive landforms (ash hills from a few meters to 10–13 m high) (Fig. 2, A) and in subordinate positions, such as defensive ditches of archaeological sites (Fig. 2, B). The soil section made in the periphery of the Borisovka site has revealed the ashy profile to a depth of 1 m, which corresponds to the mid-5th c. BC phase of the settlement. The archaeological sites of the North-West Crimea, in whose cultural strata ash has been sampled, range in date from the Late Bronze Age (second half of the 10th c. BC) to the Early Iron Age (3rd c. AD). In addition, at the two Tatarian settlements abandoned in 1944 (former villages of Saya and Ojrat) ash pits were studied. In the foothill Crimea, among the Late Scythian settlements (Ak-Kaya, Kermen-Kyr, and the village of Zayachye), Borut-Khane stands out with the ash hills ranging in height from 0.5 to 2.0 m. The earliest phase of this site dates to the 3rd–2nd c. BC, while its termination date does not exceed the 1st century AD. For the sake of ash identification, the purest layer of ash at a depth of 46–98 cm in the profile of the site's defensive earthwork was selected, which then has been sampled at a depth of 63–73 cm for chemical analyses. At present, the nearest oak woodlands are located at a distance of 4.3 km from the settlement of Borut-Khane.

Colours (dry and moist) were described using the Munsell system [3]. This system uses cylindrical coordinates, which hinders its use for statistical calculations [4]. The conversion of Munsell soil colour values into the RCIE redness index of the CIE-L*a*b* system based on a universal colour space in Cartesian coordinates was carried out using the formula [5]: RCIE = [1010 × a(a2 + b2)0.5]/(bL6). The values of parameters L, a, b were obtained from the Munsell/CIE-L*a*b* system conversion table [4]. RCIE values shown in Table 1 are for dry soil.

### 2.2 Dating of archaeological sites

The age of each site was established archaeologically (coins, amphora stamps or other narrowly datable pottery) or based on historical record [6]. Based on archaeological date of each site, the age of soils was determined. The date at which human activity at settlements ceased and pedogenesis began was controlled by the method of pedogenetic chronology, which is based on the chronosequence showing the dependence of the humus horizon thickness on soil age [7]. Ash layer from the Bronze Age and Early Iron Age site of Chernomorskoe/S11-022 (No 4) has been radiocarbon dated. A sheep/goat bone, collected for this purpose at a depth of 62 cm from the top surface, inside a 60–90 cm-thick layer of ash, has been dated to 1130–760 ВС (95.4%) (Kyiv Radiocarbon Laboratory, Ki-19342).

### 2.3 Comparison of main parent rocks and ash

The chemical composition of main parent rocks (carbonate loam and limestone eluvium) was compared with that of archaeological ash and soils formed on it (Table 2). Concentration of macro and microelements within ash deposits and soils was determined by technique of measuring metals mass fraction and oxides in powder samples using an XRF spectrometer (Spectroscan Max-GV).

As a special type of parent material, ash differs significantly in its chemical composition from the more widely distributed rocks (loam and eluvium of carbonate rocks): it has a higher content (> 30%) of P, Ca, Sr (as compared to loam) and P, Co, Sr, Zn, Pb, Cu (as compared to eluvium) and smaller concentrations of Cu, Pb, Fe, Ni (as compared to loam) and Ca (as compared to eluvium).

### 2.4 Chemical properties of soils of archaeological sites in the dry steppe

Ash pits and soils formed on ashy cultural strata were studied on the Kazantip Peninsula (Table 3), which is an area completely devoid of forests due to climatic conditions (annual precipitation 329 mm). The ash accumulation in this area stops in the 3rd century AD but the life here continued also later (7th–9th centuries AD).

The titrimetric version of the determination of C_org_ after I. V. Tyurin was carried out by oxidation of the organic substance with a solution K_2_Cr_2_O_7_ in sulphuric acid in a thermostat (at *Т* = 140 °С)), which is accompanied by the reduction of Cr (VI) to Сг^3+^. Nitrogen total (N) was determined using the Kjeldahl method (GOST 26107–84, Last Modified: 09.12.2018) “Soils. Methods for determination of total nitrogen". In titration method, nitrogen is calculated from the amount of sulphuric acid spent for titrating ammonium borate. The data obtained for C_org_ and N were used to assess the degree of enrichment of humus with nitrogen (C:N) according to the scale [8]. Acidimetric method used to determine the content of carbonates (СО_2_) in the soil is based on their destruction with hydrochloric acid solution followed by titration of its residue with sodium hydroxide solution.

### 2.5 Soil formed on ash in the forest region and in the steppe zone

A soil section on the periphery of the Borisovka settlement (forest-steppe) revealed a 1 m thick ash deposit associated with the mid-5th century BC phase of the site (Table 4). Grouping of soils and ash from archaeological sites is based on the most informative associations of chemical elements, using cluster analysis (unification by Ward's method) normalized by mean-square deviation. The chemical composition of the ash of different age from archaeological sites of North-West Crimea and of related soils is presented as an ensemble of the 12 most informative macro- and trace elements (Table 5). Data on the geochemical composition of soils and ash from the Piedmont Crimea, which are similar to objects from North-West Crimea, are presented in Table 6. Dendrogram of the ash and soil-on-ash sample distribution clearly shows two clusters of objects (Fig. 3). Table 7 summarizes the data on the geochemical composition of ash in the groups identified by the cluster analysis.

### 2.6 Main geochemical indicators of soils and ash

Table 8 shows the main geochemical indicators of the groups of objects (soils and ash) identified by the cluster analysis. The justification of the used ratios and coefficients was performed according to [10], [11], [12]. The palaeogeographic potential has an interpretation of the biogeochemical features of all soils both on defensive structures (ramparts and ditches) [13], and on the territory of settlements and in their surroundings. Using the determinations of the bulk composition of soils and ash deposits, the most informative geochemical indicators were calculated: SiO_2_/(RO + R_2_O), where RO = CaO + MgO; R_2_O = K_2_O + Na_2_O. The informative coefficient group includes the eluviation coefficient (Ke= Si/(Ca+K+Mn+Mg+Na)); coefficient of mobility (Km = (Na+K+Mg+Zn)/SiO_2_); amount of heavy metals (Σ HM = Σ (As, Co, Cr, Cu, Pb, Zn); and quality assessment for a group of chemical elements useful for plants (SQ= ∏(Ca, Al, Mn, Fe, Si, K, Mg, Ni, Cu, Zn)^1/10^). We provide a comparison of weathering coefficient (CIA values [14]) in horizon A of virgin soils so that we can compare with CIA values for soils formed on ash (Table 8). CIA values in horizon A of virgin soils vary from 26.99 (meadow steppe of the Piedmont Crimea) and 32.52 (reserved steppe of North-West Crimea) to 48.11 (coastal steppe of the Kerch Peninsula) and 59.93 (protected oak forest with an age of 250 years in Belgorod Oblast).

### 2.7 Justification of the genesis of ash deposits

The chemical composition of ash prepared from plant samples was investigated using a variety of techniques, including emission spectrometry (Zn, Cu, Mn and Co: spectrometer ICPE–9000 with induction-bound plasma), atomic absorption spectroscopy (Pb and Cd: spectrometer Quant–2AT; Hg: spectrometer Quant–Z) and photocolorimetry (As: photocolorimeter KFK–3–01). Ashing of samples was carried out in a muffle furnace at 450 °C. Comparison of the geochemical composition of oak wood ash and ash from hay of mixed grass using two ranked lists presented in Table 9. Fig. 4 shows the ratio of chemical elements in ash from archaeological sites in the forest area and sparse foothill zone. A comparison of 22 chemical elements contained in the ash from archaeological sites of the forest area (No 1) and the sparsely wooded foothill zone (No 10) has shown that these two regions used fuel of different genesis (Fig. 4). The ash obtained from the combustion of oak mixed with some other hardwoods (Borisovka settlement, mid-5th c. BC) has a higher content (> 20%) of 11 chemical elements and a lower content (<20%) of 5 chemical elements than the ash from the settlement of Borut-Khane, 1st c. BC – 1st c. AD, which is located in the region with a shortage of wood (Piedmont (forest-steppe) Crimea) and also in the arid conditions of the Roman times. Regression relationships of Zn, Cu, Mn, Cd, Pb, As, Hg, Mo, Co content in the ash from the soil section at the settlement of Borut-Khane and in the ash from various organic matter samples have made it possible to determine differences in the genesis of ash deposits.

As judged by regression lines in relation to complete junction, the ash from the settlement of Borut-Khane, 1st c. BC – 1st c. AD (Fig. 5), situated in the region which in antiquity and nowadays was short of firewood (red arrow), differs significantly from oak ash (in excess of As, Hg, Mn, Co), being most similar to the ash of feather grass (the main steppe grass) and cow dung, i.e. in composition it is close to *kizyak* which was probably the main fuel at that time.

### 2.8 Data analysis

Macro-elements and trace elements were determined by wavelength-dispersion (XRF analyser Spectroscan Max-GV). The results were quantitatively calibrated using a set of state (GOST) standard samples of soil composition. The geochemical composition of each soil sample was determined by two repeats. In case of unacceptable discrepancies between the measurements, which were detected using the spectrometer software, additional replicates were performed until an acceptable result was achieved (usually in the third repeat).

CO_2_, C_org_, total N (data presented in Table 3). C_org_ in soils was determined by the titrimetric version of Tyurin's method with oxidation in a thermostat at 140 ˚С, СО_2_ by acidometry [15]. Total nitrogen (N) was estimated by Kjeldahl's procedure. For the accuracy control, every tenth sample was measured in two repeats. In addition, after each ten measurements, a device control was carried out by measuring standard reference samples.

## CRediT Author Statement

Fedor N. Lisetskii: Conceptualization, Investigation, Writing–original draft, Funding acquisition; Arseniy O. Poletaev: Visualization; Vladimir F. Stolba: Archaeological dating, Writing–review and editing.

## Declaration of Competing Interest

The authors declare that they have no known competing financial interests or personal relationships which have, or could be perceived to have, influenced the work reported in this article.

## Data Availability

Archaeological ash deposits and soils formed on ash in the south of the East European Plain (Original data) (Mendeley Data).

## Appendix Supplementary materials

Image, application 1
